# HLA class I antibody distribution and cross-matching strategies in immune platelet transfusion refractory patients: a retrospective cohort study

**DOI:** 10.3389/fmed.2026.1839846

**Published:** 2026-08-11

**Authors:** Yu Zou, Tianhua Jiang, Ziqian Huang, Yue Yu, Mao Zheng

**Affiliations:** 1 Department of Blood Transfusion, Deyang People's Hospital, Deyang, China; 2 Department of Clinical Laboratory, Deyang People's Hospital, Deyang, China

**Keywords:** HLA class I antibodies, immune mechanism, platelet transfusion, platelet transfusion refractoriness, serological cross-matching

## Abstract

**Background:**

This study aimed to investigate the distribution characteristics and clinical significance of human leukocyte antigen class I antibodies in patients with immune-mediated platelet transfusion refractoriness (PTR) in this region, and to evaluate the clinical utility of local serological cross-matching strategies for improving platelet transfusion efficacy.

**Methods:**

A retrospective cohort study was conducted involving 229 patients who underwent platelet antibody screening. Platelet antibody screening and cross-matching were performed using solid-phase agglutination assays. Human leukocyte antigens (HLA) antibodies were detected via Luminex liquid bead array technology to obtain mean fluorescence intensity (MFI), and HLA class I antibody specificity was identified using Luminex single-antigen beads. The corrected count increment (CCI) was employed as the primary efficacy evaluation metric to compare the clinical outcomes among different transfusion strategy groups.

**Results:**

Subgroup analysis revealed: in the random transfusion group, the PTR incidence was 26.92% (7/26), with a median 24 h CCI of 7.35 (3.03, 10.78); the cross-incompatible group showed a significantly higher PTR incidence of 80.0% (4/5), with a median 24 h CCI of 0.00 (−2.54, 9.39); while the cross-compatible group had a PTR incidence of 22.72% (5/22), with a median 24 h CCI of 8.11 (3.02, 12.30). The platelet antibody positivity rate was 20.96% (48/229), with 62.5% (30/48) originating from hematologic malignancies. Among these, HLA antibody positivity reached 75.61% (31/41), with HLA class I antibodies accounting for 93.55% (29/31). HLA-B locus antibodies were the most common. The maximum MFI of HLA class I antibodies was negatively correlated with the 24 h CCI (*r*_s_ = −0.368, *p* = 0.032). The area under the receiver operating characteristic was 0.825 (95% CI: 0.600–0.954), and the optimal cut-off value was MFI = 1,525.6 (sensitivity 71.50%, specificity 85.50%).

**Conclusion:**

This study analyzed the distribution characteristics of HLA class I antibodies in suspected or highly suggestive immune-mediated PTR patients and found that the highest MFI value of HLA class I antibodies can serve as an indicator of the maximum observed HLA antibody reactivity. Based on a serological crossmatch transfusion strategy, this approach provides a feasible implementable platelet transfusion protocol for primary hospitals in southwestern China that lack HLA genotyping capabilities.

## 1 Introduction

Platelets express a complex antigen system on their surface, primarily comprising HLA, human platelet antigens (HPA), and ABH blood group antigens. Clinical observations demonstrate that immunogenic stimuli such as blood transfusion, pregnancy, or bone marrow transplantation can induce the production of platelet-related antibodies, with approximately 80% being HLA antibodies and a minority being HPA antibodies (1, 2). When antibody-positive patients receive platelets containing corresponding antigens, these antibodies can directly activate platelets through the FcγRIIa-dependent signaling pathway or facilitate rapid platelet clearance in the mononuclear phagocyte system via complement activation and membrane attack complex formation (3), his phenomenon clinically manifests as PTR.

The clinical management of immune-mediated PTR presents two major challenges: early identification/intervention and the development of effective transfusion strategies. Current research indicates that HLA antigen matching or epitope matching serves as the fundamental approach for platelet transfusion in HLA antibody-mediated PTR patients (4), but this strategy requires the support of a large donor pool. A systematic review by James M Chapman et al. (5), demonstrated that platelet cross-matching techniques can significantly improve matching efficiency to meet the clinical emergency transfusion needs. Nevertheless, whether HLA antibody specificity testing is necessary for immune-mediated PTR patients and the practical clinical value of serological cross-matching strategies still require further evidence-based medical support (6). Based on this research background, this study employed a single-center retrospective cohort design to collect clinical data from 229 patients undergoing platelet antibody screening. The study aims to establish a regionally appropriate clinical management protocol for suspected or highly suggestive immune-mediated PTR by analyzing the distribution characteristics of HLA class I antibodies and evaluating the clinical application value of cross-matching technology, thereby providing scientific evidence for developing personalized, precision transfusion strategies.

## 2 Materials and methods

### 2.1 Research population

This study employed a retrospective cohort design to systematically analyze 229 consecutive cases of patients who received platelet transfusion therapy at the Department of Blood Transfusion, Deyang People's Hospital, between September 2024 and December 2024. The subjects were recruited from multiple clinical departments, including hematology, oncology, emergency medicine, and surgery, all meeting the clinical indications for platelet transfusion. A conventional screening strategy was adopted, uniformly screening all patients requesting platelet transfusion rather than selectively screening only those with a history of transfusion, pregnancy, or clinical manifestations suggestive of PTR. Comprehensive data collection included demographic characteristics (gender, age, height, weight), hematological parameters (blood type, pregnancy history, transfusion history, history of transfusion adverse reactions), clinical diagnosis information (disease diagnosis, treatment measures, medication conditions), etc. For 48 antibody-positive patients, further HLA antibody typing results and laboratory test indicators were collected, including blood routine parameters (red blood cell count, hemoglobin concentration, white blood cell count, platelet count), platelet transfusion-related indicators (transfusion time, degree of matching, improvement of hemostatic effect), and non-immune factors that may affect the transfusion effect (fever, infection, splenomegaly, bleeding). This study was approved by the Ethics Committee of the Deyang People's Hospital (approval number: 2023-04-079-K01).

#### 2.1.1 Inclusion criteria

Patients with thrombocytopenia meeting clinical indications for platelet transfusion; Thrombocytopenic patients with an initial positive screening for platelet antibodies; Patients suspected of having platelet transfusion refractoriness due to immune factors. Platelet Antibody Screening Criteria: for patients applying for platelet transfusion for the first time, a systematic platelet antibody screening is required to assess potential immune-related transfusion risks.

#### 2.1.2 Exclusion criteria

Patients with incomplete clinical data or transfusion records; Patients who did not complete relevant laboratory tests before and after transfusion; Patients confirmed by clinical evaluation to have ineffective transfusion caused by non-immune confounding factors such as infection, fever, splenomegaly, DIC, active bleeding, and use of specific medications; Patients who completed platelet antibody testing but did not receive transfusion for various reasons.

### 2.2 Preparation and quality control of platelet products

This study utilized single-donor platelet products exclusively sourced from the Deyang Central Blood Station. All platelet preparations were processed, stored, and subjected to rigorous quality control measures in full compliance with the national standard “Quality Requirements for Whole Blood and Blood Components” (GB18469-2012). Each platelet unit contained one therapeutic dose (10U, equivalent to approximately 250–300 mL). All platelet products were prepared using the Fresenius Amicus cell separator and underwent rigorous leukocyte reduction. According to the clinical treatment protocol, all transplant recipients received γ-irradiated platelet transfusions.

### 2.3 Research methods

#### 2.3.1 Platelet antibody screening and serological cross-matching

The detection was performed using the Solid Phase Red Cell Adherence Assay (SPRCA) in conjunction with the MASPAT kit (Sanquin, batch number: 8000461039). Cross-matching was performed based on the naturally expressed HLA class I antigens (including HLA-A/B/C) on the platelet surface. During the experiment, a positive or weakly positive reaction (formation of a red blood cell layer at the bottom of the microplate well) indicated the presence of platelet-specific antibodies in the tested serum or platelet crossmatch incompatibility; a negative reaction (red blood cells aggregated at the center of the well bottom) suggested no detectable relevant antibodies and compatible matching. To ensure experimental reliability, fresh specimens from ABO-matched apheresis platelet donors collected on the same day were randomly selected for each crossmatch test, and results were interpreted based on the reaction intensity between donor and recipient.

#### 2.3.2 HLA antibody detection

The LABScreen Mixed assay (Lot No. LSM12024; One Lambda, Canoga Park, CA, USA) was utilized for the screening of HLA antibodies. Briefly, 3 mL of pre-transfusion venous blood was collected in a non-anticoagulant tube, and serum was separated via centrifugation. A total of 7 μL of patient serum was incubated with HLA Class I, Class II, and MICA mixed antigen-coated microparticles at 37 °C for 30 min. Following automated washing steps, phycoerythrin (PE)-conjugated goat anti-human IgG secondary antibody was added for a second incubation. The microspheres were then resuspended in phosphate-buffered saline (PBS), and fluorescence signals were acquired using the Luminex detection system. Data analysis and interpretation were performed using HLA Fusion software (Version 4.2; One Lambda) using the lot-specific analysis template. To eliminate non-specific background and lot-to-lot reagent variations, baseline-normalized MFI values and Normalized Background Ratios (NBG) were calculated in accordance with the manufacturer's lot-specific worksheet and our laboratory's validated Standard Operating Procedure (SOP). A sample was defined as positive if it met any of the following lot-verified criteria: (1) the highest baseline-normalized MFI of HLA Class I reactive beads > 500; (2) the highest reactive bead MFI <500 but the third-highest bead MFI > 300; or (3) the highest NBG ratio >3. For exploratory classification, a baseline-normalized MFI <500 was defined as weakly positive, and >500 was defined as positive. In this study, the highest baseline-normalized MFI value was selected as a surrogate indicator reflecting the recipient's overall anti-HLA alloimmunization profile, rather than a direct measure of donor-specific antibody (DSA) strength, given that donor HLA typing was unavailable.

#### 2.3.3 HLA class I antibody specific identification

The LABScreen Single Antigen HLA Class I kit (batch number: LS1A04014) was used for detection. The experimental steps included co-incubating the tested serum with microspheres pre-coated with specific HLA-class I antigens, then adding a fluorescently labeled anti-human IgG secondary antibody, and detecting the fluorescence intensity of each microsphere using the Luminex 3D system. Data processing was conducted with HLA Fusion 4.2 software (Beijing Mantaili Company), and baseline-normalized assignment was used to determine targeted HLA class I specificities and their corresponding alleles. Based on background-corrected and normalized detection indicators, the semi-quantitative threshold criteria for single antigen reactivity were defined as: negative (normalized MFI <500); weak positive (500 ≤ normalized MFI <5,000); positive (5,000 ≤ normalized MFI <10,000); strong positive (normalized MFI ≥ 10,000). These SAB-derived broad reaction profiles were interpreted as indicators of the recipient's sensitization breadth. It should be noted that without donor-specific typing, these results cannot directly confirm individual donor-antigen mismatches.

Among the 29 patients who tested positive for HLA Class I antibodies during the initial screening, a subgroup of 8 patients was selected for high-resolution Single Antigen Bead (SAB) assays based on the following clinical and laboratory criteria: (1) presentation of severe immune-mediated platelet transfusion refractoriness (PTR) or suspected serious transfusion reactions; (2) high-strength reactivity in the initial screening assay, defined by a peak baseline-normalized MFI > 5,000; (3) an urgent clinical requirement for virtual crossmatching to secure compatible HLA-matched platelet units.

#### 2.3.4 Quality control and interpretation criteria

(1) Instrument and Reagent Quality Control: before daily operation of the Luminex system, optical path and fluidics calibration is performed using the official beads. Each test includes built-in negative and positive controls. For the SPRCA test, if the control wells show abnormal coloration, the batch is invalidated. For the Luminex test, background subtraction is performed using negative control beads (NCB) to ensure the accuracy of baseline-normalized MFI values.
(2) Gray Zone Interpretation and Handling: to reduce the risk of false positives/negatives, a gray zone handling procedure is established: screening stage: MFI values between 300 and 500, or NBG ratios between 3 and 5, are defined as gray zone. Single Antigen Bead (SAB) stage: MFI values between 500 and 1,000 are defined as weak positive/gray zone. Handling procedure: any test result falling within the above gray zone ranges triggers duplicate retesting of the original sample, with investigation for hemolysis, lipemia, or fibrin interference. If the result remains in the gray zone after retesting, a “conservative avoidance” principle is applied in clinical matching, classifying the corresponding antigen as relatively contraindicated.
(3) Reproducibility Assessment: the laboratory regularly uses quality control sera with known sensitization levels for parallel duplicate testing. The intra-assay coefficient of variation (CV) is >10%, and the inter-assay (different test days/operators) CV is >15%, ensuring the precision and reliability of the detection system.

### 2.4 Platelet transfusion efficacy evaluation system

This study used a dual-index comprehensive evaluation of platelet transfusion effects:

(1) Laboratory index: calculate the CCI, and its formula is: CCI = [(Post-transfusion Plt count-Pre-transfusion Plt count) × 10^9^/L × BSA (*m*^2^)]/Total platelets transfused × 10^11^, where the body surface area was calculated using the modified Du Bois formula: BSA = 0.0061 × height (cm) + 0.0128 × weight (kg)−0.01529.
(2) Clinical index: evaluate the improvement of bleeding symptoms suspected or highly suggestive immune-mediated PTR (7): ABO-compatible platelet transfusion resulted in a 24-h CCI <4.5, or no significant improvement in clinical bleeding symptoms. Due to the retrospective study design and the lack of 1-h platelet count values in some cases, all study subjects used platelet count values measured 24 h after transfusion.

### 2.5 Statistical analysis methods

Statistical analysis was performed using SPSS 19.0 and GraphPad Prism 9.5.0. Baseline demographic characteristics and antibody screening positivity rates were measured with the patient as the unit of analysis, while clinical outcomes across different infusion groups were assessed with the infusion event as the unit of analysis. The specific analytical methods were as follows: (1) For continuous variables, data conforming to a normal distribution were expressed as mean ± standard deviation (x ± SD), and intergroup comparisons were conducted using the independent samples *t*-test; data not conforming to a normal distribution were expressed as median (interquartile range) [MD (Q1, Q3)], with intergroup comparisons performed using the Mann–Whitney *U*-test. (2) Categorical variables were reported as absolute and relative frequencies [*n* (%)],” and intergroup comparisons were performed using the χ^2^ test, for small sample subgroups (e.g., mismatched group, *n* = 5), Fisher's exact test was used for statistical inference. (3) To account for the non-independence of multiple platelet transfusion events in the same patient, generalized estimating equations (GEE) were employed to analyze clinical outcomes across different infusion groups, including the incidence of PTR and the 24 h-CCI. (4) Correlation analysis: Shapiro–Wilk test was performed to assess the normality of continuous variables. Both HLA class I MFI and 24 h CCI did not follow normal distribution, so Spearman rank correlation analysis was adopted to evaluate the monotonic correlation between the two indicators. (5) This study used HLA class I antibody MFI as the core independent variable, incorporating clinical non-immune characteristics such as age, sex, pregnancy history, transfusion history, clinical diagnosis, and the presence of fever or infection as covariates. With the binary outcome of PTR as the dependent variable, a multivariate binary logistic regression model was constructed. Based on the joint prediction probability output by the model, a receiver operating characteristic (ROC) curve was plotted, and the model's discriminative ability was evaluated by calculating the area under the curve (AUC). The optimal cutoff value, sensitivity, and specificity were determined according to the maximum Youden index. All statistical tests were two-sided, with the significance level set at *p* < 0.05.

## 3 Results

### 3.1 Study population and clinical characteristics

**Figure 1:**
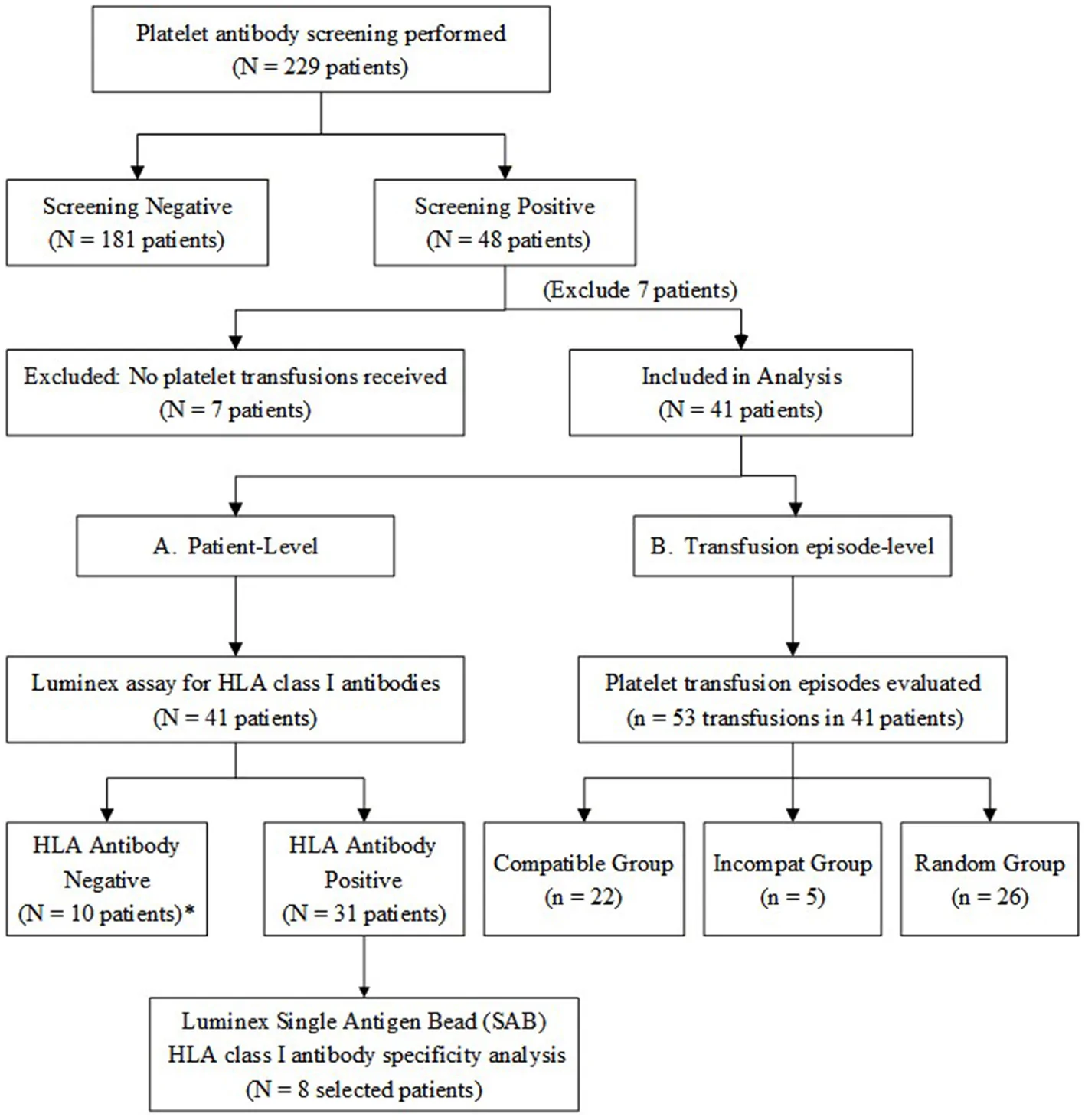
Flowchart of platelet antibody screening and cross-matching procedures. “Patient-level analyses” (using patient count *N* as the denominator) “transfusion episode-level analyses” (using transfusion count *n* as the denominator).

The platelet antibody positivity rate was 20.96% (48/229), with 62.5% (30/48) of positive cases originating from hematologic malignancies. Among the 48 antibody-positive patients, 74 platelet transfusion requests were made, with 53 transfusions actually administered to 41 patients. Among the 41 patients, 31 patients received a single transfusion episode, 8 patients received two episodes, and 2 patients received three episodes, totaling 53 transfusion episodes. The PTR incidence was 30.19% (16/53), among whom 10 exhibited bleeding symptoms such as subcutaneous petechiae and ecchymosis, but the improvement in bleeding symptoms did not meet the expected clinical standards. Subgroup analysis showed that the incidence of PTR in the random transfusion group was 26.92% (7/26), with a median 24-h CCI of 7.35 (3.03, 10.78); in the crossmatch-incompatible group, the PTR incidence was significantly higher at 80.00% (4/5), with a median 24-h CCI of 0.00 (−2.54, 9.39); while in the crossmatch-compatible group, the PTR incidence was 22.72% (5/22), with a median 24-h CCI of 8.11 (3.02, 12.30). This study included 53 platelet transfusion events, and the generalized estimating equation (with an exchangeable correlation structure) was used to adjust for the correlation of repeated transfusions in the same patient, as well as to control for confounding factors such as baseline diagnosis, pre-transfusion infection/fever, and baseline bleeding score to mitigate indication bias. Pairwise comparisons of PTR risk between groups are as follows:

1. The crossmatch-incompatible group compared to the compatible group: adjusted OR = 9.12, 95% CI (1.01, 82.45), *p* = 0.048, indicating that crossmatch incompatibility is an independent risk factor for PTR.
2. The random transfusion group compared to the crossmatch-compatible group: adjusted OR = 1.43, 95% CI (0.41, 5.03), *p* = 0.612, showing no statistically significant difference in PTR risk between these two groups.
3. The crossmatch-incompatible group compared to the random transfusion group: adjusted OR = 7.65, 95%CI (0.92, 63.17), *p* = 0.061, indicating no statistically significant difference between groups after adjusting for clinical confounding factors. Detailed comparison results between groups are shown in Table 1. Figure 1 illustrates the workflow for platelet antibody testing and crossmatch procedures in the study population.

**Table 1:** Analysis of 53 platelet transfusion episodes in 41 patients with immune-mediated platelet transfusion refractoriness.

Index parameters	Cross-matching compatible group (*n*)	Cross-matching incompatible group (*n*)	Random transfusion group (*n*)
Number of transfusions (*n*)	22	5	26
Frequency (%)	43.08	10.77	46.15
24 h CCI (median, IQR)	8.11 (3.02, 12.30)	0.00 (−2.54, 9.39)	7.35 (3.03, 10.78)
PTR incidence (%)	22.72% (5/22)	80.00% (4/5)	26.92% (7/26)

Cross-matching compatible group: priority is given to transfusing platelet products that have been cross-matching and found compatible; Random transfusion group: due to platelet inventory limitations or urgent clinical needs, ABO-matching but non-cross-matching random platelets are transfused; Cross-matching incompatible group: ABO-matched but cross-incompatible platelets are transfused. Some critically ill patients are in severe condition and cannot find matched platelets in a short time, so clinicians strongly request the transfusion of random or cross-matching incompatible platelets.

### 3.2 Serological cross-matching and transfusion efficacy analysis

This study systematically evaluated 27 cross-matched platelet transfusions in 25 patients, with comprehensive laboratory parameter assessments conducted pre- and post-transfusion. As demonstrated in Table 2, platelet count emerged as the sole parameter exhibiting statistically significant improvement (*p* < 0.05). The core efficacy evaluation indicators for platelet transfusion are platelet count and 24-h CCI, with no significant changes observed in red and white blood cell counts. This further confirms that the variations in platelet counts in this study are specific effects of platelet transfusion, unaffected by the infusion of other blood components or significant interference from non-immune factors. During the 76 donor screening procedures performed for these 27 cross-matching events, the positive cross-match rate was 46.05% (35/76), while the negative rate was 53.95% (41/76). Statistical analysis revealed that each patient required an average of 3.04 platelet cross-matching procedures (76/25), with each cross-matching event necessitating screening of 2.81 potential donors on average (76/27).

**Table 2:** Laboratory parameters before and after 27 cross-matched transfusions in 25 patients.

Parameter	Pre-transfusion (*n* = 27)	24 h Post-transfusion (*n* = 27)	*t*-value	*p*-value
RBC count (× 10^12^/L)	2.56 (2.08, 2.95)	2.54 (2.03, 2.81)	−1.977	*p* = 0.059
Hemoglobin (g/L)	72.50 (65.00, 86.50)	73.00 (63.00, 81.00)	−1.771	*p* = 0.088
WBC count (× 10^9^/L)	2.76 (0.88, 9.09)	2.38 (0.84, 9.92)	0.996	*p* = 0.329
Platelet count (× 10^9^/L)	14.00 (7.50, 23.25)	26.00 (15.00, 32.00)	5.289	*p* < 0.05

RBC, red blood cell; WBC, white blood cell. The study cohort comprised 25 patients with diverse hematological disorders, including 14 cases of acute leukemia, 5 cases of thrombocytopenia, 1 case of myelodysplastic syndrome, 1 case of severe aplastic anemia, 1 case of multiple myeloma, and 3 cases of other hematologic conditions.

### 3.3 Analysis of HLA antibody detection results

This study systematically investigated HLA antibody profiles in 41 patients diagnosed with platelet alloimmunization. Comprehensive immunological screening demonstrated a significant HLA antibody positivity rate of 75.61% (31/41), with a notable gender distribution showing female predominance (64.52%, 20/31) compared to males. Among them, there was one female patient with no history of pregnancy, 11 with one pregnancy, and 8 with two or more pregnancies. Detailed characterization of HLA antibody specificities showed that 93.55% (29/31) of HLA-positive patients exhibited HLA class I antibodies, with remarkably broad antibody strength spectrum (normalized MFI range: 749.15–16,479.96). As presented in Table 3, these findings strongly suggest that HLA class I antibodies constitute the predominant immunogenic factor in suspected or highly suggestive immune-mediated PTR, highlighting their critical role in alloimmune platelet destruction.

**Table 3:** Distribution patterns of HLA antibody reactivity in study cohort.

Antibody class	Strong positive (*N*)	Weak positive (*N*)	Negative (*N*)
HLA Class I	19	10	2
HLA Class II	12	6	13
MICA	1	6	24

We performed two statistical comparison strategies between HLA Class I and Class II: Three ordered tiers (strong positive, weak positive, negative): pearson's χ^2^ test showed no statistically significant difference in full reactivity distribution (χ^2^ = 5.050, *p* = 0.080).

Binary grouping after combining all positive samples: two-sided Fisher's exact test revealed that the overall sensitization positive rate of HLA Class I was significantly higher than HLA Class II (*p* = 0.028).

### 3.4 Distribution patterns of HLA class I antibody specificities

Patient-level profiling of the eight subselected individuals revealed a highly complex landscape of HLA alloimmunization (Table 4). All eight patients demonstrated multi-specific reactivity against various HLA Class I allele-coated beads (MFI ≥ 500). High-strength sensitization was predominant at the patient level, with 6 out of 8 patients (75.00%) harboring potent antibody responses with a peak MFI ≥ 10,000. In this patient cohort, seven patients (87.50%) showed concurrent sensitization at multiple loci. Among them, three patients (37.50%) exhibited extensive simultaneous reactions to microspheres associated with the A, B, and C loci.

**Table 4:** Allelic distribution of HLA class I specific antibodies (MFI ≥ 500) in eight patients.

HLA-A locus antibodies			HLA-B locus antibodies			HLA-C locus antibodies		
Allele	Frequency (*n* ≥3)	Prevalence (%)	Allele	Frequency (*n* ≥3)	Prevalence (%)	Allele	Frequency (*n* ≥1)	Prevalence (%)
A^*^66:02	5	62.5	B^*^13:02	6	75	C^*^02:02	4	50
A^*^23:01	4	50	B^*^40:01	6	75	C^*^18:02	2	25
A^*^02:01	3	37.5	B^*^07:02	5	62.5	C^*^06:02	2	25
A^*^02:03	3	37.5	B^*^08:01	5	62.5	C^*^15:02	2	25
A^*^02:06	3	37.5	B^*^15:02	5	62.5	C^*^04:01	2	25
A^*^24:02	3	37.5	B^*^27:05	5	62.5	C^*^07:02	2	25
A^*^24:03	3	37.5	B^*^27:08	5	62.5	C^*^03:03	1	12.5
A^*^25:01	3	37.5	B^*^40:02	5	62.5	C^*^03:02	1	12.5
A^*^26:01	3	37.5	B^*^73:01	5	62.5	C^*^03:04	1	12.5
A^*^34:02	3	37.5	B^*^78:01	4	50	C^*^12:03	1	12.5
A^*^43:01	3	37.5	B^*^15:10	4	50	C^*^01:02	1	12.5
A^*^66:01	3	37.5	B^*^49:01	4	50	C^*^16:01	1	12.5
A^*^26:01	3	37.5	B^*^35:01	4	50	C^*^14:02	1	12.5
			B^*^15:11	4	50	C^*^05:01	1	12.5
			B^*^45:01	4	50	C^*^17:01	1	12.5
			B^*^44:03	4	50			
			B^*^42:01	4	50			
			B^*^41:01	4	50			
			B^*^13:01	4	50			
			B^*^81:01	4	50			
			B^*^48:01	4	50			
			B^*^40:06	4	50			
			B^*^47:01	4	50			

### 3.5 Correlation analysis between maximum MFI value of HLA class I antibodies and platelet transfusion efficacy (*N* = 34)

**Figure 2:**
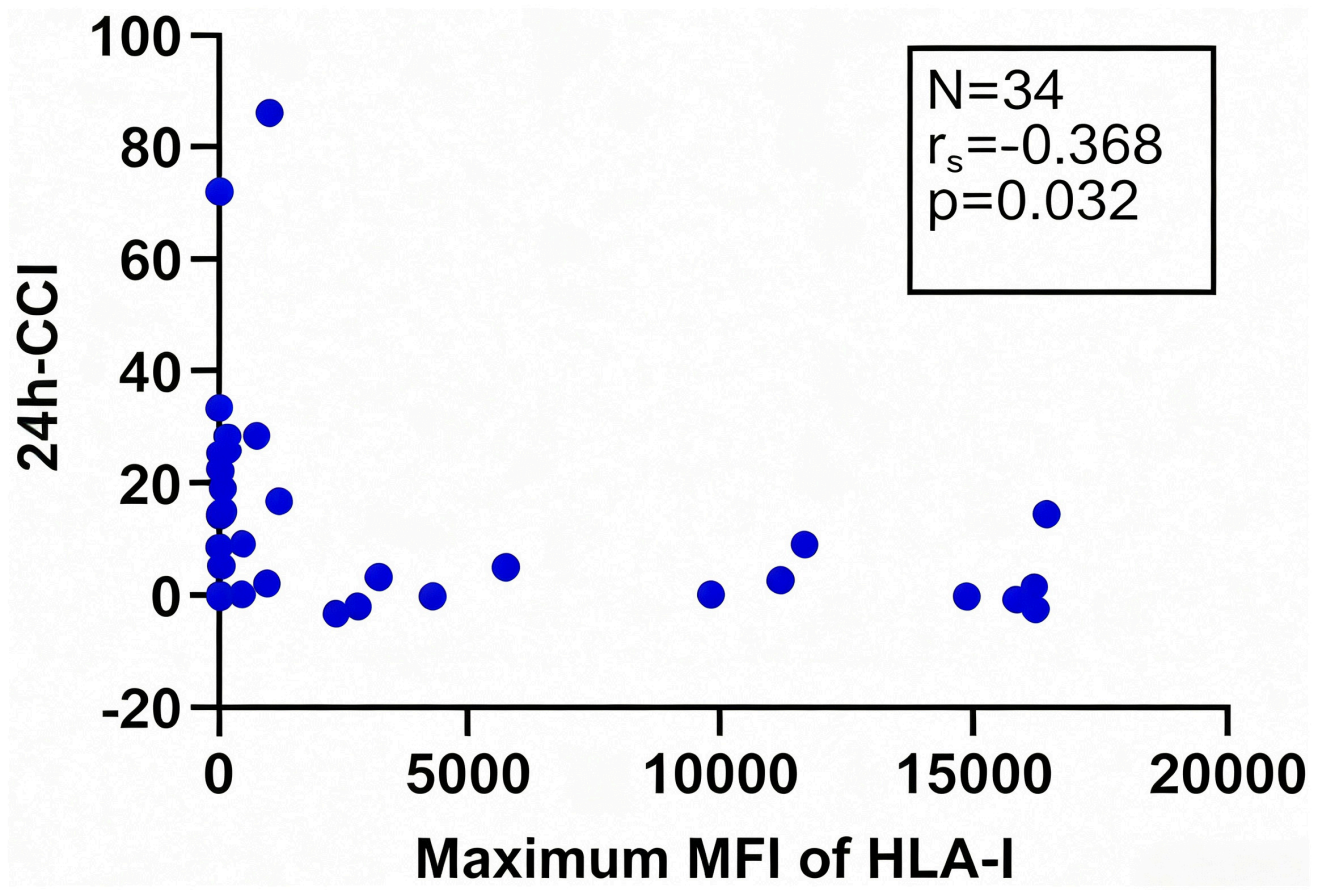
Spearman's rank correlation analysis between the maximum MFI value of HLA class I antibodies and the 24-h CCI (*N* = 34). *N* = 34 is analyzed from the patient level, excluding repeated infusion events.

Using Spearman's rank correlation analysis, the results showed a significant negative monotonic correlation between the maximum MFI value of HLA class I antibodies and the 24-h CCI (*r*_s_ = −0.368, *p* = 0.032). This finding suggests that an increase in the MFI level of HLA class I antibodies is closely associated with a decrease in platelet transfusion efficacy (Figure 2). This negative correlation further indicates that, in this patient population, higher antibody levels may adversely affect the clinical response to platelet transfusion.

### 3.6 Predictive efficacy of the highest MFI value of HLA class I antibodies combined with multiple factors on platelet transfusion outcomes (*N* = 34)

**Figure 3:**
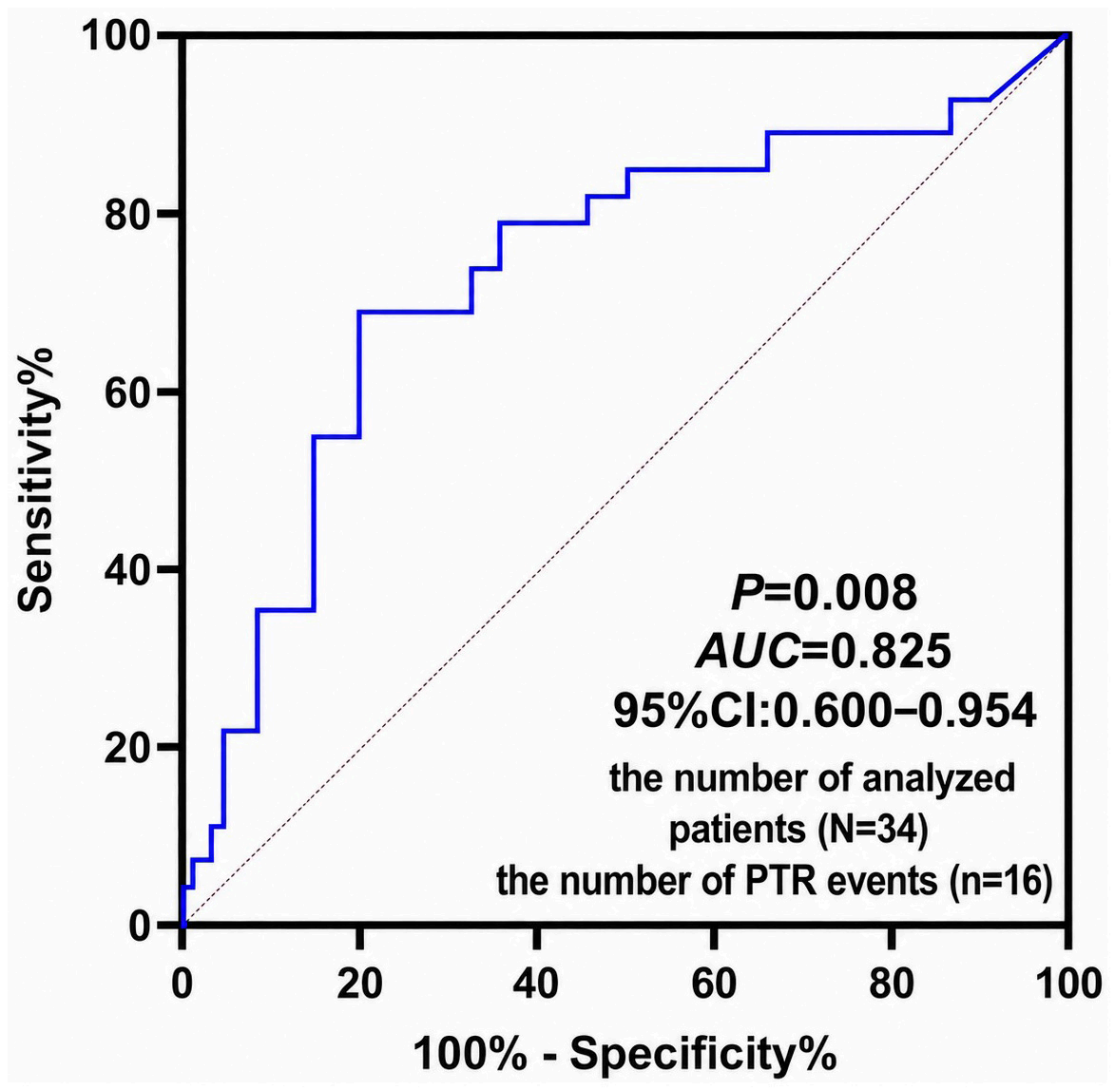
Predictive efficacy of HLA class I antibody maximum MFI on platelet transfusion outcomes: a multivariable ROC curve analysis (*N* = 34). *N* = 34 is the number of analyzed patients. *n* = 16 is the number of PTR events.

This study constructed a multivariate binary logistic regression model, using the highest MFI value of HLA class I antibodies as the core independent variable, while incorporating clinical non-immune characteristics such as age, sex, pregnancy history, transfusion history, clinical diagnosis, and fever/infection as covariates. The combined predictive probability output by the model was used as the plotting indicator to construct a ROC curve (Figure 3), aiming to provide evidence-based support for clinical decision-making in immune platelet transfusion refractoriness. The results showed that the AUC of this multivariate combined prediction model was 0.825 (95% confidence interval: 0.600–0.954, *p* < 0.05). The optimal predictive probability cutoff value determined based on the maximum Youden index was 1,525.6. At this threshold, the combined predictive sensitivity of the model was 71.50%, specificity was 85.50%, and the maximum Youden index was 0.566.

## 4 Discussion

Platelet transfusion serves as a critical therapeutic intervention for thrombocytopenia and related hemorrhagic disorders. However, its efficacy is frequently compromised by immunological factors. Extensive research has demonstrated that repeated platelet transfusions can induce the production of various platelet antibodies, leading to PTR. Among the immunological mechanisms underlying PTR, HLA class I antibodies emerge as the predominant pathogenic factor due to their high polymorphism, followed by HPA antibodies, ABO blood group antibodies, and CD36 (GPIV) antibodies (7). Notably, the genetic polymorphism of HLA-A and HLA-B loci predisposes recipients to develop HLA class I antibodies during multiple transfusion episodes, establishing this as the fundamental immunological basis for clinical PTR (8, 9). Current clinical management of immune-mediated PTR primarily employs selective platelet transfusion strategies (10, 11), with core techniques including: HLA-matched platelet transfusion, donor specific antibody (DSA)-avoidance transfusion, Platelet cross-matching. These approaches enhance transfusion safety and efficacy by achieving immunological compatibility between donors and recipients, thereby reducing alloimmunization rates. However, the limited scale of HLA genotype databases for platelet donors in most Chinese blood centers has rendered serological cross-matching the routine detection method in current clinical practice. This situation underscores the urgent need for in-depth investigation of HLA antibody distribution patterns and optimization of cross-matching techniques.

This study involved a consecutively enrolled cohort from routine clinical screening, which minimized selection bias to the greatest extent. The antibody positivity rate of 20.96% reflects the true antibody distribution characteristics among platelet transfusion patients in this region, which is relatively high, likely because the study population predominantly consisted of hematology and oncology patients (62.50% from hematology departments). This aligns with previous research (12) reporting elevated platelet antibody positivity in patients with hematological malignancies and solid tumors. Among antibody-positive patients, the PTR incidence was 30.19%, aligning with multiple domestic and international studies (9, 13, 14). These results further corroborate platelet antibodies as significant risk factors affecting transfusion outcomes, a conclusion supported by Song X et al.'s meta-analysis (15). All single-donor platelets used in this region undergo leukocyte reduction, a current practice that may lower the risk of alloimmunization in patients. Seftel MD et al. (16) demonstrated that universal leukoreduction of blood components significantly reduced PTR incidence from 40 to 23% (*p* < 0.05). Similarly, Gilson and Zimring (17) established that removing donor antigen-presenting cells effectively mitigates alloimmunization risks in immunocompromised patients through both direct and indirect recognition pathways.

The results of this study demonstrate that the transfusion efficacy of crossmatch-compatible platelets using SPRCA is superior to that of the crossmatch-incompatible group (*p* < 0.05), markedly improving the CCI value, which aligns closely with relevant research outcomes (18–20). Univariate Fisher's exact test without confounder adjustment revealed that PTR incidence was markedly higher in the cross-match incompatible group, but such disparity was confounded by critical clinical conditions such as active infection and severe bleeding. After multivariate GEE adjustment, only cross-match incompatibility remained significantly associated with elevated PTR risk compared with cross-match compatible platelets. We acknowledge that the allocation of patients to the random or crossmatch-incompatible groups was partly driven by clinical urgency and inventory constraints, introducing potential indication bias. Although multivariate adjustments for infection, fever, and disease severity were performed, these findings should be interpreted cautiously as exploratory evidence of serological matching benefits in real-world settings. Notably, during chemotherapy-induced myelosuppression, certain patients showed minimal platelet count improvement despite receiving crossmatch-compatible products, followed by spontaneous recovery post-chemotherapy. This observation suggests that chemotherapy-induced bone marrow suppression may play a crucial role, though the underlying mechanisms warrant further investigation.

This study focuses on HLA class I antibody distribution patterns and crossmatching strategies in suspected or highly suggestive immune-mediated PTR patients, addressing a research gap regarding HLA antigen distribution characteristics in Southwest China. Our results demonstrate a remarkably high HLA antibody prevalence of 75.61%, closely aligning with previous reports (62%) (21), but significantly exceeding the 40% reported by Salama OS et al. (20), potentially reflecting regional HLA distribution peculiarities. Among the 20 female patients with positive HLA antibodies, with 95% (19/20) of female patients having a history of pregnancy, while all 11 male patients with positive HLA antibodies had a clear history of cumulative blood product exposure or prior stem cell transplantation, explaining the source of their alloimmune response. A noteworthy finding was the significantly higher proportion of female patients (64.52%) developing alloimmunization compared to males (*p* < 0.05). This result is consistent with Kleinman S et al.'s findings (22) that 17% of female blood donors and 24% of parous women exhibited detectable HLA antibodies. This phenomenon likely stems from fetal HLA antigen exposure during pregnancy, where leukocyte fragments enter maternal circulation and induce specific antibody production (23, 24). HLA class I antibody subtype analysis revealed a 93.55% detection rate among positive cases, concordant with existing literature (25–27). Notably, the distribution of these positive microsphere reactions displayed significant patient heterogeneity and a pronounced clustering pattern of cross-reactive groups (CREGs). The multi-bead reactions were not independent immune events but were mediated by antibodies in the recipients targeting specific “shared epitopes” or public epitopes. Analysis indicated that the primary targeted epitopes were concentrated on cross-reactions involving the HLA-B locus and the HLA-A locus, while specific reactions targeting the HLA-C locus were relatively limited in both breadth and intensity in most patients (7, 28).

Cross-matching analysis revealed that among 25 PTR patients undergoing 27 matching procedures involving 76 donor samples, the positive rate reached 46.05%, which aligns with findings reported by Kingsley S (29). Notably, our study demonstrated lower matching frequencies (3.04 times per patient) and fewer donors per matching (2.81 samples) compared to previous literature (20), potentially reflecting platelet donor resource scarcity in the Deyang region. These findings suggest that in primary hospitals lacking molecular diagnostic techniques, solid-phase red cell adsorption crossmatching remains a feasible and accessible alternative triage strategy.

Spearman rank correlation analysis revealed a significant negative correlation between the maximum MFI value of HLA class I antibodies and 24-h CCI (*r*_s_ = −0.368, *p* = 0.032), indicating that higher HLA class I antibody titers may lead to poorer platelet transfusion outcomes. The multivariate logistic regression combined prediction model demonstrated good predictive performance, with an AUC of 0.825. The MFI cut-off value of 1,525.6 identified in this study represents an exploratory threshold, consistent with the findings of Linjama T et al. (21), where MFI > 1,000 is an independent risk factor for platelet transfusion refractoriness, but lower than the 3,000 MFI threshold reported by Blandin L et al. (30). However, due to the lack of external validation, this threshold may overestimate diagnostic performance. Before it can be recommended as a clinical standard, this threshold must be validated in larger, independent multicenter cohorts. These findings collectively establish HLA class I antibody MFI values as an indicator of the maximum observed HLA antibody reactivity that may overestimate diagnostic performance due to lack of external validation. This threshold must be validated in larger, independent, multicenter cohorts before it can be recommended as a clinical standard. Consequently, for HLA class I antibody-positive patients, implementing platelet cross-matching or HLA-matched platelet transfusion represents an effective strategy for PTR prevention (31, 32). These results provide compelling evidence to support the establishment of regional platelet donor registries.

The innovation of this study is mainly reflected in the following three aspects: first, by systematically analyzing HLA antibody characteristics in a patient population from Southwest China, we found that the highest MFI value of HLA class I antibodies can serve as an indicator of the maximum observed HLA antibody reactivity. Second, through in-depth analysis of HLA class I antibody specificity distribution patterns and MFI value fluctuations, we discovered that multi-locus sensitization may correlate with PTR severity. Finally, the methodological framework established in this study has strong clinical applicability and potential for generalization, providing valuable references for similar research in other regions. Based on our findings, we propose the following recommendations: (1) Hematology and oncology departments should implement HLA class I antibody specificity testing for patients requiring repeated platelet transfusions, prioritizing platelets lacking the corresponding antigens; (2) Establishment of compatible platelet donor screening mechanisms to prevent the development of HLA class I antibodies; (3) Development of regional HLA gene databases to provide personalized platelet transfusion protocols for long-term transfusion recipients and highly sensitized patients, thereby optimizing platelet resource allocation.

This study has several limitations. First, it adopted a single-center retrospective design with a relatively limited sample size, which may affect the statistical power. Second, the method used to determine the MFI threshold in this study is exploratory in nature and requires validation through independent samples. Additionally, due to the lack of CCI assessment data immediately after transfusion (within 10–60 min), it is difficult to fully distinguish between immune and non-immune factors, and the superimposed effects of non-immune factors cannot be completely ruled out. Finally, constrained by the scale of the local HLA gene database and the absence of donor HLA typing data, donor-specific antibody (DSA) analysis could not be conducted. It is recommended that future multicenter collaborative studies with larger sample sizes be carried out to more comprehensively reveal the distribution characteristics of HLA class I antibodies in this region.

## 5 Conclusion

This study elucidates the distribution characteristics of HLA class I antibodies in patients with suspected or highly suggestive immune-mediated PTR, and finds that the highest MFI value of HLA class I antibodies can serve as an indicator of the maximum observed HLA antibody reactivity. In addition, the compatibility of platelet cross-matching is significantly associated with higher transfusion efficiency and CCI values, providing a feasible and accessible platelet transfusion strategy for primary hospitals in the southwestern region that lack HLA genotyping capabilities.

## Data Availability

The raw data supporting the conclusions of this article will be made available by the authors, without undue reservation.
